# Supplementary material for: Investigating structural and functional aspects of the brain’s criticality in stroke
Source: Sci Rep. 2023 Jul 31;13:12341. doi: 10.1038/s41598-023-39467-x (PMC10390586; doi:10.1038/s41598-023-39467-x)
Supplement: Supplementary file 1 — Supplementary Information. [file 41598_2023_39467_MOESM1_ESM.pdf]

Supplementary Information to *Investigating structural and functional aspects of the brain's criticality in stroke*

Jakub Janarek[1,+], **Zbigniew Drogosz**[1,+], **Jacek Grela**[1,2,+], **Jeremi K. Ochab**[1,2,*], **and Paweł Oświęcimka**[1,2,3]

[1]Institute of Theoretical Physics, Jagiellonian University, 30-348 Kraków, Poland
[2]Mark Kac Center for Complex Systems Research, Jagiellonian University, 30-348 Kraków, Poland
[3]Complex Systems Theory Department, Institute of Nuclear Physics, Polish Academy of Sciences, 31-342 Kraków, Poland
[*]jeremi.ochab@uj.edu.pl
[+]These authors contributed equally to this work.

ABSTRACT

This paper addresses the question of the brain's critical dynamics after an injury such as a stroke. It is hypothesized that the healthy brain operates near a phase transition (critical point), which provides optimal conditions for information transmission and responses to inputs. If structural damage could cause the critical point to disappear and thus make self-organized criticality unachievable, it would offer the theoretical explanation for the post-stroke impairment of brain function. In our contribution, however, we demonstrate using network models of the brain, that the dynamics remain critical even after a stroke. In cases where the average size of the second-largest cluster of active nodes, which is one of the commonly used indicators of criticality, shows an anomalous behavior, it results from the loss of integrity of the network, quantifiable within graph theory, and not from genuine non-critical dynamics. We propose a new simple model of an artificial stroke that explains this anomaly. The proposed interpretation of the results is confirmed by an analysis of real connectomes acquired from post-stroke patients and a control group. The results presented refer to neurobiological data; however, the conclusions reached apply to a broad class of complex systems that admit a critical state.

1 Investigation of various measures of criticality

We calculate an extended list of criticality measures for the empirical connectomes of stroke patients and for the critical and non-critical human-connectome-based Watts-Strogatz networks. This Supplementary Information serves as an extension to Fig. 1 where the three most relevant quantities were shown. The results of this extended study are reported in Supplementary Fig. 1.

2 Other modifications of the models

In this section, we apply different modifications to the Ising and Haimovici models to demonstrate the robustness of the main result presented in Fig. **??**. We swap the modifications in a complementary way. We gradually disconnect subsystem B in the Ising model (in the main text, we varied the subsystem size), and we vary the subsystem size in the Haimovici model by disconnecting different single RSNs (in the main text, we gradually disconnected only the auditory RSN). Both results are shown in Supplementary Fig. 2. In this study, the Ising model runs on a 32×32 lattice and subsystem B is a patch of size 16×16.

In the Ising case, Supplementary Fig. 2**a**, we find a family of curves forming a degenerate comb with its upper non-critical branch consisting of a single line corresponding to all connections removed. The remaining modifications cover the lower branch of the comb and exhibit indicators of criticality. This behavior is characteristic of the model in which even a single connection between the subsystems is sufficient to connect the clusters and make the behavior qualitatively the same as in an unmodified system.

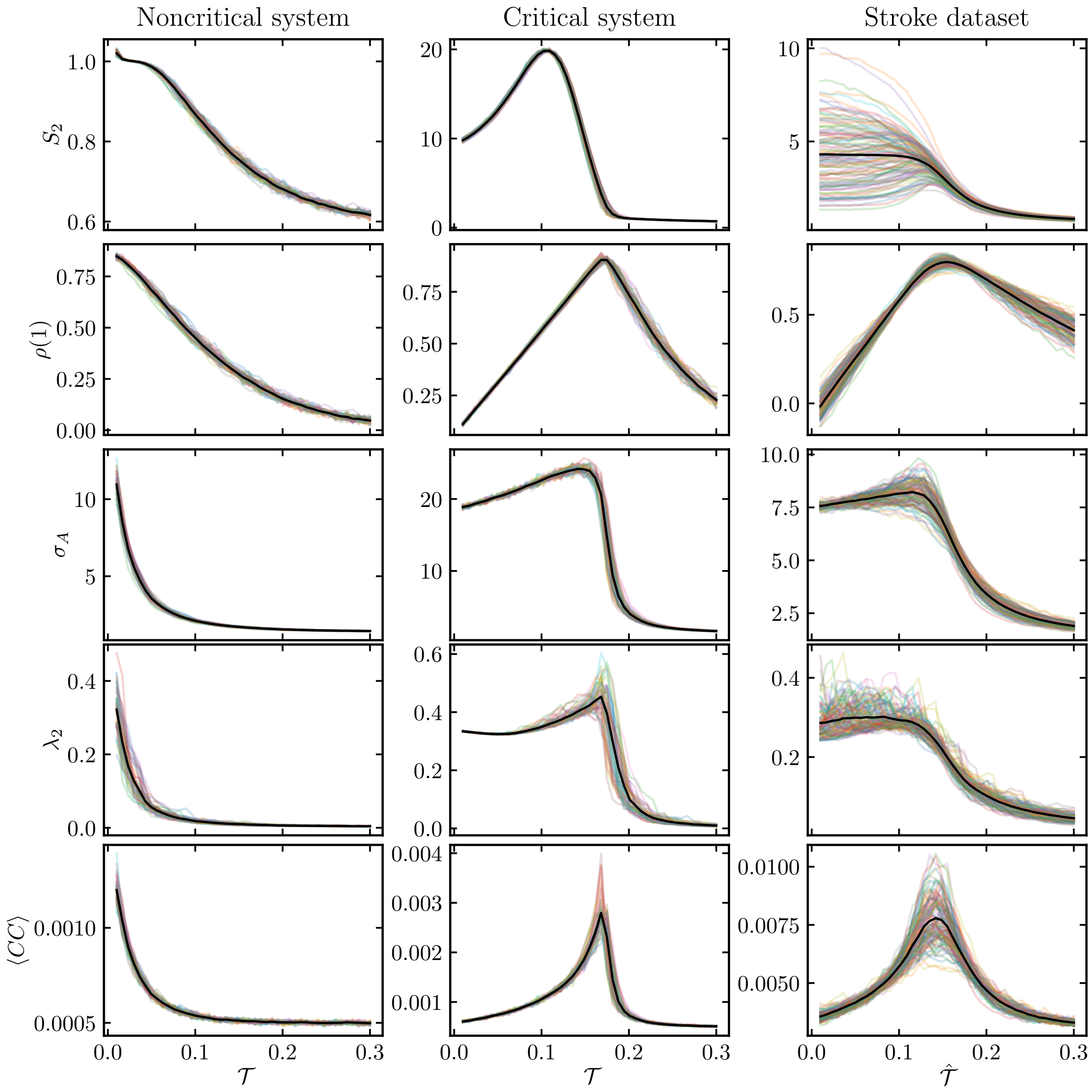

Figure 1. Criticality measures in the Haimovici model. Left and middle: noncritical and critical connectome-based Watts-Strogatz networks. **Right:** stroke dataset connectomes. The measures include: S_2, the size of the second largest cluster of activity; $\rho(1)$, the first coefficient of the autocorrelation function; σ_A, the standard deviation of the total activity; λ_2, the second largest eigenvalue of the node activity cross-correlation matrix; $\langle CC \rangle$, the average of cross-correlation matrix elements.

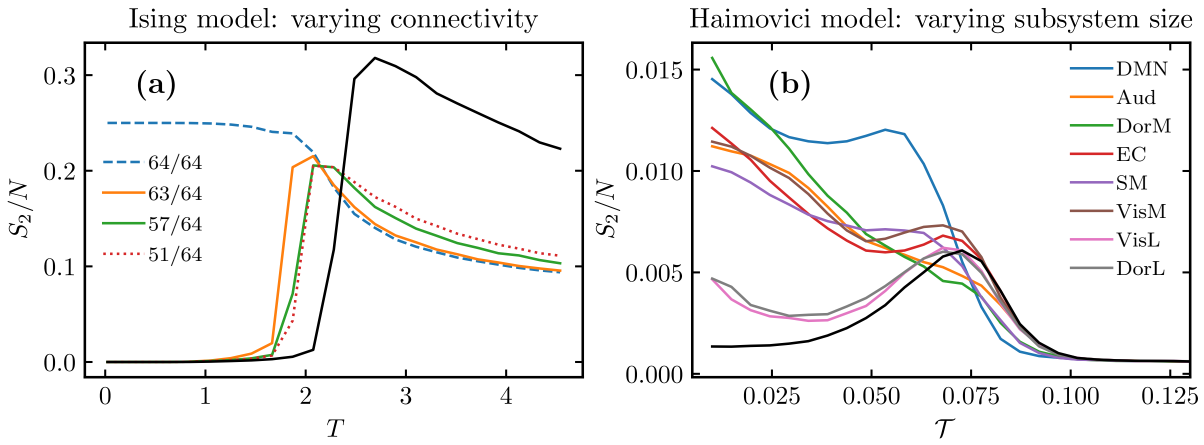

Figure 2. Dependence of the size of the second largest cluster, S_2, on: **(a)** temperature for various fractions of removed connections between the subsystems in the Ising model on a lattice of size 32×32 with the smaller subsystem defined as a 16×16 patch, and **(b)** the threshold parameter for fully disconnected RSN of varying sizes and locations in the Haimovici model. The legend is ordered from the smallest resting-state network *DorL* (46 nodes) to the largest *DMN* (128 nodes). Black lines denote cluster sizes S_2 for unmodified systems. These plots supplement Fig. **??** by inspecting other ways of modifying the systems than those described in the main text.

Disconnecting different RSNs serves as a proxy for varying the size of the subsystem. In this case, the resulting family of curves does form a comb-like pattern, albeit somewhat perturbed; this is not a surprise, as different brain substructures are not rescaled copies of each other. Still, the curves are size-ordered to a certain degree. The most prominent examples of partitions that preserve the critical peak (such as *DorL* and *VisL*) are the smallest RSNs, and those that appear non-critical (such as *DMN*) are the largest ones. This is in agreement with the Ising case with varying subsystem size, as seen in Fig. **??a**. The competition between S_2^A and S_1^B and the resulting second largest cluster of the entire system when individual RSNs are fully disconnected is additionally depicted in Supplementary Fig. 3.

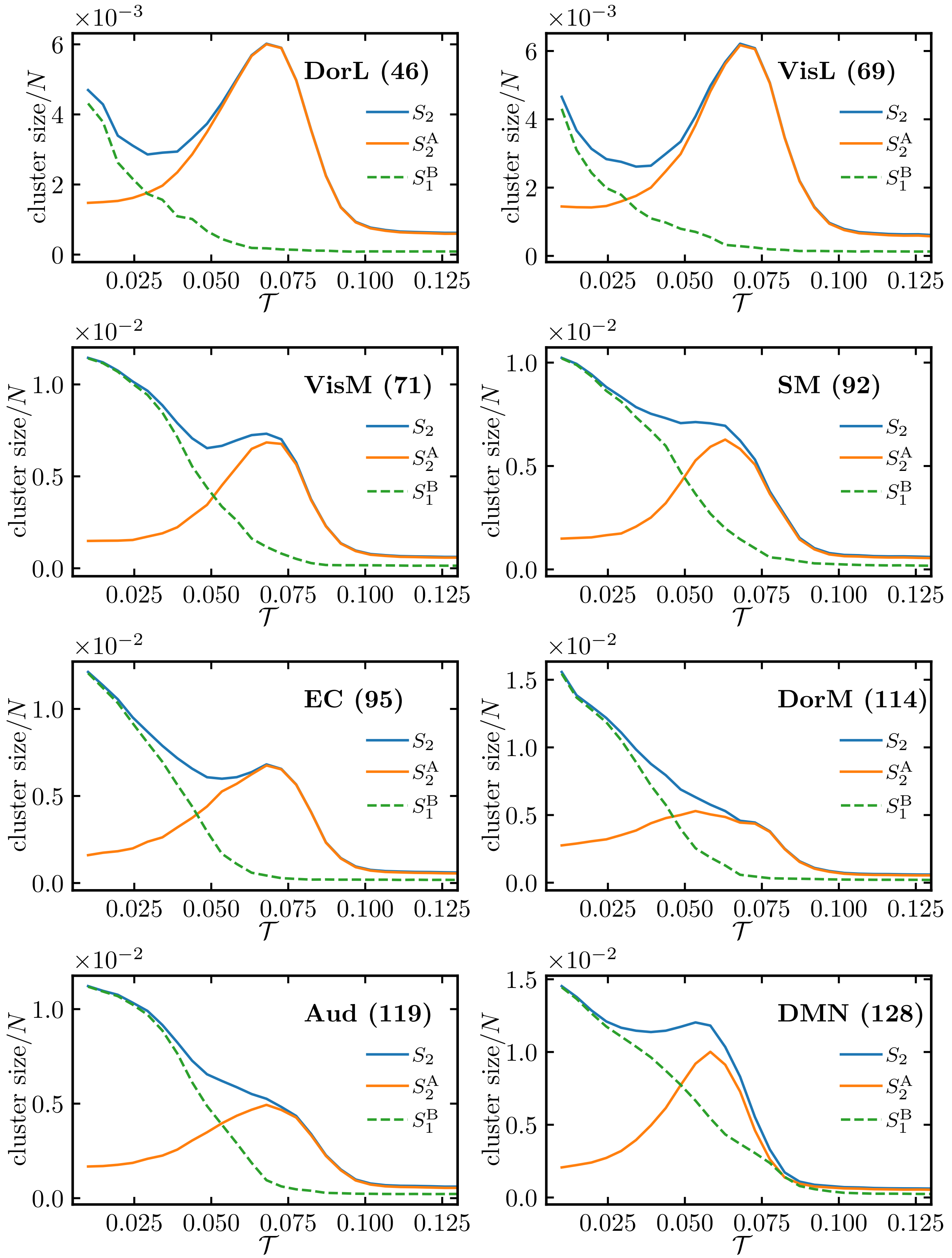

Figure 3. Cluster sizes in the Haimovici model for the Hagmann et al.'s connectome with various RSNs fully disconnected from the rest of the network. Each panel shows the second largest cluster of the entire system, S_2 (blue line), the second largest cluster of the larger subsystem, S_2^{A} (orange line), and the largest cluster of the disconnected RSN (abbreviation and size give in the panel), S_2^{B} (dashed green line). In all the cases, at some value of the threshold parameter $\mathcal{T}$, the order of sizes changes, impacting the size of the second largest entire-system cluster. Depending on the case, the peak in S_2 may persist (e.g., *DorL*, *VisL*) or be absent (e.g., *DorM*, *Aud*, and *DMN*).
